# Systematic analysis of the regulation of type three secreted effectors in *Salmonella enterica *serovar Typhimurium

**DOI:** 10.1186/1471-2180-7-3

**Published:** 2007-01-18

**Authors:** Yakhya Dieye, Jessica L Dyszel, Rebin Kader, Brian MM Ahmer

**Affiliations:** 1 Biodesign Institute, Arizona State University, Tempe, AZ 85287 USA; 2 Department of Microbiology, The Ohio State University, Columbus, OH 43210 USA

## Abstract

**Background:**

The type III secretion system (TTSS) is an important virulence determinant of Gram-negative bacterial pathogens. It enables the injection of effector proteins into the cytosol of eukaryotic cells. These effectors ultimately manipulate the cellular functions of the infected organism. *Salmonella enterica *serovar Typhimurium encodes two virulence associated TTSSs encoded by the *Salmonella *Pathogenicity Islands (SPI) 1 and 2 that are required for the intestinal and systemic phases of the infection, respectively. However, recent studies suggest that the roles of these TTSSs are not restricted to these compartments. The regulation of TTSSs in *Salmonella *is very complex with several regulators operating to activate or to repress expression depending on the environmental conditions.

**Results:**

We performed a systematic analysis of the regulation of type III effectors during growth in vitro. We have tested the ability of seven regulatory genes to regulate ten effector genes. Each regulator was expressed in the absence of the other six to avoid cascade effects. Our results confirm and extend the previously reported regulation of TTSS1 and TTSS2 effectors by InvF-SicA and SsrB respectively.

**Conclusion:**

The set of strains constructed for this study can be used to quickly and systematically study the regulation of newly identified effector genes of *Salmonella enterica*. The approach we have used can also be applied to study complex regulatory cascades in other bacterial species.

## Background

The type III secretion system (TTSS) is a complex molecular machine found in numerous Gram-negative bacterial pathogens of animals and plants [1]. This secretion system encodes a syringe-like organelle that injects effector proteins directly into the cytosol of eukaryotic cells. The effectors ultimately affect host cell physiology.

**Figure 1:**
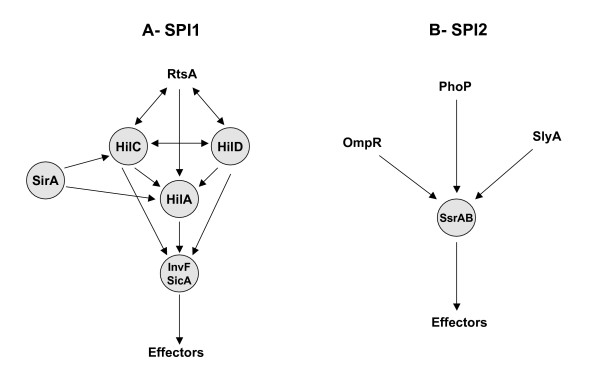
Regulatory cascades activating the expression of SPI1 (A) and SPI2 (B) related type III effectors. The regulators analyzed in this study are represented in circled boxes.

*S. enterica *serovar Typhimurium (hereafter referred to simply as Typhimurium) possesses two virulence associated TTSSs encoded by the *Salmonella *Pathogenicity Islands (SPI) 1 and 2 [2]. TTSS1 (encoded by SPI1) delivers effectors that mediate the invasion of epithelial cells and the intestinal events of Typhimurium infection [3-5]. TTSS2 is required during the systemic phase of Typhimurium infection [6]. It secretes effectors that allow the survival and multiplication of the pathogen within macrophages [7]. The regulation of the Typhimurium TTSSs is complex with several regulatory proteins involved (Figure 1). SPI1 encodes five regulators, four of which are known to be involved in regulatory cascades that lead to the expression of genes inside and outside of SPI1 [8]. The central regulator of SPI1 gene expression is HilA, a member of the OmpR/ToxR family [5,8]. HilA directly activates the expression of two SPI1 operons that encode TTSS1 apparatus components [5,9]. One of these operons begins with the *invF *gene, which encodes a transcriptional activator of the AraC family. InvF activates the expression of TTSS1 effectors encoded both inside and outside of SPI1 [10,11]. The activity of InvF requires the SicA co-regulator which is also encoded within SPI1 [12,13]. The third and fourth regulators are HilC and HilD, both of which belong to the AraC family. Both can directly activate the expression of *hilA *[14,15]. They can also activate the expression of the *invF *operon independently of HilA [16,17]. The fifth regulator encoded within SPI1, SprB, contains a LuxR/UhpA helix-turn-helix motif, however no target genes for SprB have been identified [18].

SPI1 gene expression is also regulated by factors encoded outside of SPI1 (Figure 1A). The SirA/BarA two-component system is an ancient regulatory system with orthologs found throughout the γ-proteobacteria that is involved in virulence gene expression, exoenzyme and antibiotic production, motility, and biofilm formation [19,20]. In Typhimurium, SirA has been shown to bind and activate the promoters of *hilC *and *hilA *[21], although an alternate model has also been proposed in which SirA acts through *hilD *rather than through *hilA *and *hilC *[22]. Additional regulators of SPI1 gene expression encoded outside of SPI1 include the two-component regulatory systems PhoPQ [23], PhoBR [24], and OmpR/EnvZ [24], and the AraC-like transcriptional activator RtsA [25]. How these regulatory inputs are integrated is not yet known.

The central regulator of SPI2, and some TTSS2 effector genes located outside of SPI2, is the SsrAB two-component system [26]. SsrB has been shown to bind and activate the promoter of the *srfH/sseI *gene that encodes a TTSS2 effector [27]. Similarly, SsrB is thought to directly activate the expression of SPI2 operons as well as other effector genes located outside of SPI2 [28,29]. The expression of *ssrA *and *ssrB *is activated by factors encoded outside of SPI2 (Figure 1B). These include the two-component systems OmpR/EnvZ [30], PhoPQ [31], and the *slyA *gene [32]. Recently the *ydgT *gene was identified as a negative regulator of SPI2 gene expression [33].

The effectors secreted by TTSS1 are required for the invasion of intestinal epithelial cells [4,34]. In contrast, the SPI2 genes are induced after Typhimurium has invaded or is phagocytized by eukaryotic cells [29,31,35-37]. These observations led to the hypothesis that TTSS1 is needed to invade intestinal cells, but is not required during the subsequent phases of Typhimurium pathogenesis, while TTSS2 is expressed only when the bacteria reside within eukaryotic cells. Several recent reports suggest a more complicated role of the Typhimurium TTSSs. For example, SPI1 mutants have a replication defect and are unable to synthesize a normal SCV inside epithelial cells suggesting that SPI1 genes are involved in these functions [38]. Conversely, SPI2 genes have been shown to be involved in the induction of the inflammatory response caused by *S. enterica *serovar Dublin in a bovine ligated-ileal-loop model [39] and to be essential for the full virulence of Typhimurium in murine infectious enterocolitis [40,41]. These results suggest that each TTSS might be expressed in different compartments within the host, and that at least some effectors might be involved in more than one phase of the infection.

Given the complexity of these regulatory hierarchies, we took a systematic in vitro approach to categorizing the regulatory inputs of effector genes in Typhimurium. Besides examining the effects of individual regulatory mutations on individual effectors, we wanted to test each regulatory gene in the absence of other regulators. For example, the regulatory hierarchy for SPI1 is complex in that SirA activates *hilA *and *hilC*, HilC also activates *hilA*, and HilA and HilC in turn activate *invF*. InvF combined with SicA then activates effector genes. Since mutations anywhere in the cascade can cause loss of effector gene expression, we chose to test individual members of the cascade for activation of each effector in the absence of all other members of the cascade. This type of approach could reveal if, for instance, *sirA, hilA*, or *hilC *can regulate any effectors independently of *invF*. This approach might also reveal novel regulatory patterns for individual effectors, for example, effector genes regulated by both SPI1 and SPI2 regulators. Therefore, in this report we have examined the effects of seven regulatory genes on ten effector genes. The expression of each effector gene was examined in a set of strains lacking all but one of the seven regulators. Our results confirm and extend the known regulation of TTSS1 and TTSS2 effectors by *invF-sicA *and *ssrB *respectively.

## Results and discussion

### Construction of Typhimurium multi-regulator mutant strains harboring *lacZY *fusions to type III effector genes

We constructed a Typhimurium strain that lacks seven regulators, six of which are known to affect the expression of the type III secretion systems of this bacterium. The TTSS1-encoding region of SPI1, which harbors five regulatory genes (*hilC, hilD, hilA, invF *and *sprB*) and a co-regulator (*sicA*), was deleted in a strain that harbors a mutation of the *sirA *gene (see Materials and Methods). Transduction of an *ssrB *mutation into this strain resulted in YD038 which lacks seven regulatory genes (hereafter known as the multi-mutant). Chromosomal *lacZY *transcriptional fusions to ten effector genes were then constructed in YD038. These included fusions to genes that encode effectors secreted by TTSS1 (*sopA, sopB *and *sopE2*), TTSS2 (*sspH2, sifA, sifB, sseI*, and *sseG*) or both (*slrP *and *sspH1*). These ten fusions were also transduced into the wild type strain, 14028, using phage P22HTint.

### Effects of individual regulators on the expression of the type III secreted effectors

**Figure 2:**
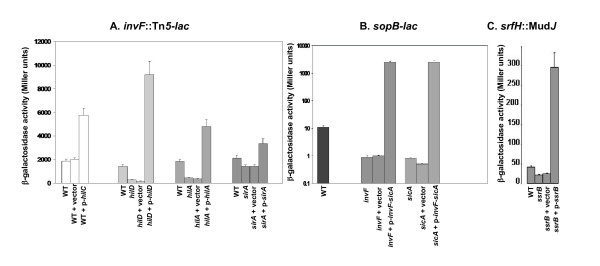
Functional verification of regulator-encoding plasmids. Each regulatory gene, cloned into the pWSK29 vector, was introduced into a strain containing a mutation of the corresponding regulator and a *lacZY *reporter to a gene known to be activated by the regulator (The *hilC*-encoding plasmid was introduced into the wild type strain and compared to the control vector). β-galactosidase assays were performed on log-phase cultures (using ONPG) and the results represent a mean of at least three independent assays. **(A) **An *invF*::Tn5-*lacZY *reporter was used to verify the plasmids encoding *hilA, hilC, hilD *and *sirA*. **(B) **A *sopB-lacZY *fusion was used for the plasmid encoding *invF *and *sicA*; and **(C)**, a *srfH*::Mud*J *reporter for the plasmid encoding *ssrB*.

Low copy number plasmids individually encoding seven regulators of SPI1 or SPI2 (or the vector control) were transformed into each of the ten strains that carry a *lacZY *fusion in the multi-mutant background. Each plasmid was constructed in such a way as to have the regulatory gene expressed from the vector's *lac *promoter. Expression and regulatory activity of the resulting constructs was confirmed using complementation tests with known target genes (Figure 2). The only exception was *sprB *for which the target genes are not known. The resulting 80 strains (and the ten additional strains harboring the same fusions in the wild-type background) were then grown in triplicate to log phase under either SPI1-inducing conditions (LB broth standing at 37°C overnight [42]) or SPI2-inducing conditions (MgM broth shaking at 37°C until OD_595 _of 0.2 to 0.8). The β-galactosidase activity of the strains was then determined. The results of SPI1-inducing conditions are shown in Table 1 and the results of SPI2-inducing conditions are shown in Table 2. For all of the discussion below, any effect less than 3-fold is considered to be no effect.

#### • TTSS1 effectors

Regardless of growth conditions, the fusions to *sopA, sopB*, and *sopE2 *produced more β-galactosidase in the wild type than in the multi-mutant vector control suggesting that a regulatory factor is missing in the multi-mutant (Tables 1 and 2). For *sopA *the *invF-sicA *plasmid was the only plasmid that restored expression. Expression of the *sopB *fusion was also restored by the *invF-sicA *plasmid. These results confirm previous findings in which *invF-sicA *activates *sopA *and *sopB *[10,25]. Interestingly, the *ssrB *plasmid also partially restored *sopB *expression, but only in SPI1-inducing conditions. This is a novel result but the effect of *ssrB *is quite small compared to the effect of *invF *so it is not clear if it is physiologically relevant.

The expression of the *sopE2 *fusion was decreased in the multi-mutant background and the *invF-sicA *plasmid restored expression (Tables 1 and 2). The activation of *sopE2 *by *invF-sicA *has not been previously reported. However, a homolog of *sopE2*, the *sopE *gene, has been shown to be regulated by *invF-sicA *[12]. The *sopE2 *gene is present in all *Salmonella *serovars examined to date, while the presence of *sopE *is sporadic [43]. The *sopE *gene was not included in this study because it is not present in our wild-type 14028 strain. Our results suggest that in addition to their similar function, *sopE *and *sopE2 *are both regulated by *invF-sicA*.

Additionally, all of the above results indicate that the regulators above *invF *in the SPI1 regulatory cascade (*sirA, hilA, hilC, hilD*) cannot regulate *sopA, sopB*, or *sopE2 *in the absence of *invF*.

#### • *slrP *and *sspH1*

SspHl and SlrP are known to be secreted by both TTSS1 and TTSS2 [28,44]. The *sspH1 *fusion was not decreased in the multi-mutant background compared to the wild-type in either SPI1 or SPI2-inducing conditions, suggesting that none of the seven regulators control *sspH1*. However, the *invF-sicA *plasmid activated the *sspH1 *fusion by 4-fold compared to the vector control in SPI1-inducing conditions (Table 1). Previously Miao and Miller found that the expression of *sspH1 *is constitutive [44], so it is not clear if the 4-fold effect of a plasmid-encoded regulator is physiologically significant.

Expression of the *slrP *fusion did not decrease in the multi-mutant background in either growth condition, but it was activated 12-fold and 4-fold in the presence of the *sprB *and *invF-sicA *plasmids, respectively. This effect was only seen in SPI1-inducing conditions. Both of these results are interesting since no target has been identified for *sprB*, and *invF-sicA *was not known to regulate *slrP*. It has been reported that while chromosomal *hilC *and *hilD *have no effect on *slrP*, plasmid-encoded *hilC *and *hilD *do moderately activate *slrP *expression [25]. In this study, the *slrP *fusion was induced neither by *hilC *nor by *hilD *in the multi-mutant background suggesting that the previously observed regulation of *slrP *by *hilC *and *hilD *is through *invF*.

**Figure 3:**
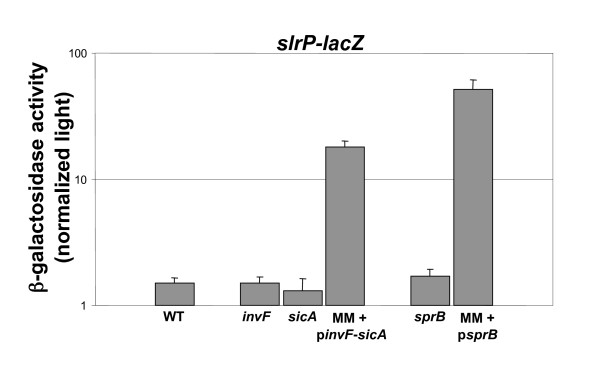
Effect of chromosomal regulators on the expression of *slrP*. β-galactosidase assays were performed on log-phase cultures (using Beta-glo reagent). The results represent a mean of at least three independent assays. WT, wild type; invF, *invF *mutant; sicA, *sicA *mutant; sprB, *sprB *mutant, MM + pinvF-sicA, multi-mutant containing the plasmid encoding *invF *and *sicA; *MM + psprB, multi-mutant containing the plasmid encoding *sprB*.

To test the activation of the *slrP *fusion by chromosomal *sprB *and *invF-sicA*, mutations in these regulators were introduced into an otherwise wild-type background carrying a chromosomal *slrP-lacZY *fusion. None of the mutations affected the expression of the *slrP *fusion (Figure 3). This is consistent with the lack of a decrease in the multi-mutant background and a previous report in which plasmid-encoded *rtsA, hilC*, and *hilD *had effects on *slrP*, but little or no difference in expression was seen with the same regulators expressed from the chromosome [25].

#### • TTSS2 effectors

Regardless of growth condition, four of the five fusions to TTSS2 effector genes (*sspH2, sifB, sseI*, and *sseG*) were activated by the *ssrB*-encoding plasmid (Tables 1 and 2). This confirms the known activation of these genes by the *ssrAB *two-component system [29,44,45]. Expression of the *sifA *fusion was not affected in the multi-mutant background compared to the wild-type, regardless of growth condition. Additionally, none of the plasmid-encoded regulators altered *sifA *expression. This result was unexpected since the expression of *sifA *has previously been shown to depend on *ssrA*, which encodes the sensor kinase component of the SsrAB two-component system [44].

To try to reconcile this discrepancy, we constructed a second chromosomal *lac *fusion to *sifA *using the method developed by Ellermeier *et al*. (see Methods). This fusion was moved into the wild type and the multi-mutant backgrounds. The multi-mutant strain was then transformed with the *ssrB*-encoding plasmid or the pWSK29 vector. This *sifA *fusion was not regulated by *ssrB *under SPI1-inducing conditions and was only regulated 2-fold under SPI2-inducing conditions (Table 3). SifA expression was almost twice as high in the wild-type strain as it was in either the Δ*ssrA*, Δ*ssrB*, or Δ*ssrAB *strains. Furthermore, *sifA *expression was 2-fold higher in the multi-mutant background containing the *ssrB *plasmid than it was in the vector control strain (Table 3).

**Figure 4:**
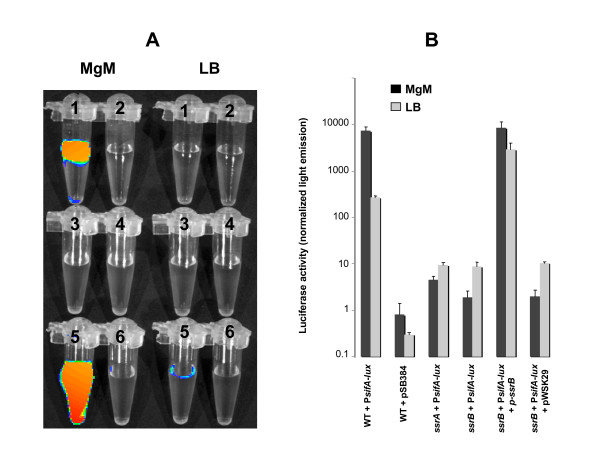
*ssrB *activates the promoter of *sifA*. The expression of the *luxCDABE *operon under the control of the P*sifA *promoter was analyzed in the wild type and the isogenic *ssrA *mutant and *ssrB *mutant with or without complementation. **A**, the strains were grown in MgM or in LB for six hours, and the light emitted by bacterial suspensions of similar cell density were captured using the Xenogen IVIS imaging system (Alameda, CA) and displayed as pseudocolor images with blue representing the lowest and red the highest light intensity. 1, wild type + P*sifA-lux*; 2, wild type + pSB384 vector control; 3, *ssrA *+ P*sifA-lux*; 4, *ssrB *+ P*sifA-lux*; 5, *ssrB *+ P*sifA-lux *+ *pssrB*; 6, *ssrB *+ P*sifA-lux *+ pWSK29 control. **B**, quantification of the activation of the P*sifA *promoter. The light emitted by 100 microliters of culture was measured using a luminometer. The values represent the mean ± SEM of three independent experiments.

To test the regulation of *sifA *by *ssrB *in another way, we constructed a plasmid that harbours the *luxCDABE *operon from *Photorhabdus luminescens *under the control of the *sifA *promoter. This plasmid was placed into the wild type, the *ssrA *mutant, and the *ssrB *mutant containing either the p*ssrB *plasmid or the vector control. These strains were grown in LB or MgM media and the light emitted by the cells captured using a Xenogen IVIS imaging system (Alameda, CA) (Figure 4A) and measured using a luminometer (Figure 4B). The light emitted by the wild type strain is higher in MgM compared to LB showing that the P*sifA *promoter is induced in the MgM medium (Figure 4). The activity of the P*sifA *also decreased strongly in the *ssrA *and the *ssrB *mutants after growth in both MgM and LB. The p*ssrB *plasmid restored the production of light in the *ssrB *mutant (Figure 4). These results are consistent with previous findings that *sifA *is regulated by the *ssrAB *two-component regulatory system. It is not clear why the regulatory effects are seen so clearly with plasmid-encoded fusions and not observed with chromosomal fusions. It is possible that additional layers of regulation are imposed on the chromosomal fusions such as supercoiling or chromatin structure that are relieved on the plasmid.

Expression of the *sifB *fusion was activated by *ssrB*, as expected [29,44,45]. However, *sifB *was also repressed by *sirA, hilC*, and *invF*. This is an interesting and novel result because a TTSS2 effector is being repressed by the SPI1 regulatory cascade. No other effectors are activated by one system and repressed by the other. Even more unusual is that *sirA *and *hilC *had their effects in the multi-mutant background lacking *invF*. This indicates that *sirA *and *hilC *are acting on *sifB *independently of the standard SPI1 regulatory pathway.

## Conclusion

We have taken a systematic approach to study the regulation of type III effector genes during growth in vitro. Our results confirm that *sopA *and *sopB *are activated by *invF-sicA*. In addition we have determined that *sopE2 *and *sspH1 *are regulated by *invF-sicA*. The regulation of *sopE2 *is very strong under either SPI1 or SPI2-inducing conditions while the regulation of *sspH1 *is weak and only observed under SPI1-inducing conditions. The *sspH2, sifB, sseI *and *sseG *fusions were shown to be strongly activated by *ssrB*. The most interesting regulation was observed with *sifA *and *sifB*. Chromosomal fusions to *sifA *were largely unaffected by any regulator. However, a plasmid-based *sifA *fusion was strongly regulated by *ssrB*. The *sifB *fusion was strongly regulated by *ssrB*, as expected. However, it was also strongly repressed by *sirA, hilC*, and *invF*. This is the first indication that *sirA *and *hilC *might regulate an effector gene independently of *invF*. The set of strains constructed for this study can now be used to quickly and systematically study the regulation of newly identified effector genes.

## Methods

### Growth conditions, enzymes, reagents, and transduction

The strains and plasmids used in this study are described in Additional file 1. The bacterial strains were grown in LB (Luria-Bertani) medium at 37°C. For induction in the Magnesium minimal medium (MgM) [46] cells from LB overnight cultures were washed with MgM prior to 1:50 dilution. The following antibiotics were obtained from Sigma and used at the following concentrations when required: kanamycin (kan, 50 μg/ml), ampicillin (amp, 100 μg/ml), chloramphenicol (cam, 20 μg/ml), tetracycline (tet, 20 μg/ml), and hygromycin (hyg, 200 μg/ml). Sucrose was supplemented at 219 mM final concentration to select against *sacB*. The β-galactosidase chromogenic substrate 5-bromo-4-chloro-3-indolyl-β-D-galactopyranoside (X-Gal) was obtained from Molecular Probes (Eugene, OR) and used at a final concentration of 40 μg/ml.

General molecular biology techniques were performed essentially as described [47]. Restriction and modification enzymes were purchased from Invitrogen (Carlsbad, CA) or New England Biolabs (Beverly, MA), and used as recommended by the manufacturers. PCR primers were purchased from IDT Inc. (Coralville, IA). Plasmids were extracted using kits from Qiagen (Valencia, CA). P22 transduction was performed as described [48].

### β-galactosidase assay

The β-galactosidase activities were measured using o-nitrophenyl β-D-galactopyranoside (ONPG, Sigma, St. Louis MO) using the method described by Miller [49] or the Beta-Glo™ assay system (Promega, WI) according to the manufacturer's recommendations. Briefly the assay consisted of mixing an equal volume of a log phase growing culture (OD_590 _0.2–0.8) and a detection reagent. The latter contains (i) a luciferin-galactoside substrate (6-O-β-galactopyranosyl-luciferin) that is cleaved by the β-galactosidase to form luciferin and galactose, and (ii) a firefly luciferase that catalyzes the luciferin to generate light. After at least 1 hour of incubation at room temperature the light produced is measured in a Turner Designs TD-20/20 luminometer or a Wallac 1420 VICTOR^3 ^plate reader and the results expressed as light per OD_595 _per μl of culture. The results were expressed as the mean ± SD of at least three different experiments.

### Mutation of regulatory genes

The *invF*, *sicA*, *sprB*, *ssrA*, and *ssrB *genes were replaced with the *cat *cassette from pKD3 using the λ-red recombination system as described [50].

### Construction of a multi-regulator mutant strain

The TTSS genes of SPI1 (~35 kb), but not the manganese transport genes [51], were deleted using the pRE112 suicide vector [52]. This deleted *avrA *through *invH*. To do this, PCR was performed using primers BA1093 and BA1094 (see Additional file 2) with 14028 genomic DNA as template. The 1366 bp product contains the 3' end of the *invH *gene, *stm2901*, and the 5' end of *stm2902*. A second PCR was performed using primers BA1095 and BA1096 (see Additional file 2). The 1255 bp product contains the 3' end of *sitC*, the *sitD *gene, and the 5' part of the *avrA *gene. Both products were cloned into pCR2.1 Topo (Invitrogen). The *sitD*-containing fragment was removed with *Sac*I and cloned into the *Sac*I site of the *stm2901*-containing clone. This placed the two fragments adjacent to each other and created the Δ(*avrA-invH*)1 deletion. This 2894 bp fragment was removed with *Kpn*I and *Pvu*II and cloned into the suicide vector pRE112 that had been digested with *Kpn*I and *Sma*I to yield the plasmid pYD25. This plasmid contains (i) a *cat *gene that confers a resistance to chloramphenicol, (ii) the R6K replication origin that requires the *pir *gene, and (iii) the *sacB *gene of *Bacillus subtilis *that is toxic in Gram-negative bacteria grown in the presence of high concentrations of sucrose [53]. Allelic exchange was performed by mobilizing pYD25 from BW20767 into JVR140 (*sirA4*::*hyg*). The chloramphenicol resistant transconjugants harbor the pYD25 plasmid integrated into the chromosome, resulting in the duplication of the region upstream and downstream of TTSS1. Isolated colonies were grown in LB without chloramphenicol selection until the exponential phase and plated onto LB lacking NaCl and containing 219 mM sucrose to select for loss of the integrated plasmid by homologous recombination. The colonies obtained were screened for Δ(*avrA-invH*)1 using PCR with primers hybridizing upstream and downstream of the TTSS1 region. Finally, the resulting Δ(*avrA-invH*)1 *sirA4*::*hyg *strain was used as a recipient for the P22HTint transduction of an *ssrB*::*cat *mutation from MJW129 [29] to make the multi-mutant YD038 strain. YD038 lacks the co-regulator *sicA*, and seven regulators, *sirA, ssrB, hilA, hilC, hilD, invF*, and *sprB*.

### Construction of *lacZY *fusions to effector genes

To make *lacZY *transcriptional fusions to effector genes, the 3' portion of each gene (including the stop codon but without the transcription terminator) was amplified using PCR and cloned into pCR2.1 TOPO (Invitrogen, Carlsbad, CA). These gene portions were then removed as *Eco*RI fragments and cloned into the *Eco*RI site upstream of the promoterless *lacZY *gene of the pVIK112 *pir*-dependent suicide vector [54]. The resulting plasmids were mobilized from BW20767 into YD038. The correct integration of the plasmids into the chromosome of the resulting kanamycin-resistant transconjugants was verified by PCR. Fusions were transduced using phage P22HTint into other backgrounds as needed. A second fusion to *sifA *was constructed as described previously [55]. First the lambda red recombination system was used to integrate a kanamycin resistance gene flanked by FRT (Flp recombinase target) sites from the pKD4 plasmid [50]. The FRT-kan-FRT cassette was placed after the stop codon, but before the transcription terminator, of *sifA*. Second, the temperature sensitive pCP20 plasmid [56] that encodes Flp recombinase was introduced into the strain. Flp-mediated excision of the *kan *cassette left a single FRT site that serves as an integration point for the fusion. The third step was to integrate the pCE36 suicide plasmid that has a FRT site directly upstream of the promoterless *lacZY *[55]. After the integration of pCE36, the pCP20 plasmid was cured by growth at 37°C.

### Construction of plasmids encoding regulators of the *Salmonella *type III secretion systems

Each of the regulatory genes missing in the multi-mutant was amplified using PCR (the primers used are described in Additional file 2), cloned into the pCR2.1 TOPO vector, released by enzymatic digestion and cloned under the control of the *lac *promoter of pWSK29. The *hilD, sirA*, and *hilA *genes were released from the TOPO vector as 1353 bp *Sac*I-*Xba*I, 936 bp *Kpn*I-*Xho*I, and 1920 bp *EcoR*I fragments respectively and cloned into the corresponding sites of pWSK29 to yield the plasmids pYD17, pYD28 and pYD29 respectively. The 971 bp *Spe*I-*Xho*I fragment containing the *sprB *gene was cloned into the *Xba*I-*Xho*I sites of pWSK29 to give the pYD15 plasmid. The *hilC *gene was released as a 1541 bp *Pvu*II-*Xba*I fragment and ligated to the *Eco*RV-*Xba*I sites of pWSK29 to produce the plasmid pYD16. A 852 bp *Eco*RI fragment and a 622 bp *Kpn*I-*Xho*I fragment containing the *invF *and the *sicA *genes were cloned into the corresponding sites of pWSK29 to yield the plasmids pYD23 and pYD38 respectively. The 5353 bp *Apa*LI-*Xho*I fragment from pYD23 was ligated to the 1527 *Apa*LI-*Xho*I fragment from pYD38 to give the pYD40 plasmid that harbours the *invF *and *sicA *genes as a bi-cistronic operon under the control of the *lac *promoter. Each construction was verified by the complementation of a corresponding regulatory mutant (Figure 2), except for *sprB *for which a complementable function has not yet been discovered [18]. We sequenced both strands of the *P_lac_*-*sprB *region of the *sprB*-encoding plasmid to ensure that the sequence was correct.

### Construction of the P*sifA-lux *reporter plasmid

The promoter region and the 5'part of the *sifA *gene was PCR-amplified from 14028 genomic DNA using the primers YD1 and YD2 (see Additional file 2). The product was cloned into the pCR2.1 TOPO vector (Invitrogen) and then released as an *Eco*RI fragment that was subsequently cloned upstream of the *luxCDABE *operon of the pSB384 vector [57]. The correct orientation of the P*sifA *promoter in the resulting pYD56 plasmid was verified by PCR.

### Cell imaging and luminometry

Liquid cultures were grown in LB or MgM media for six hours. The cultures were brought to similar OD_600 _and 200 μl of cell suspensions were placed in tubes and imaged using the Xenogen IVIS imaging system (Alameda, CA). The images are displayed as pseudocolors with blue representing the lowest and red the highest light intensity. For luminometry the light emitted by 100 μl of culture was measured using a Turner Designs TD-20/20 luminometer.

## Authors' contributions

BMMA provided the original idea for this study. YD designed and conducted the experiments. RK and JLD participated in the molecular biology and enzymatic assays. YD and BMMA wrote the article. All authors read and approved the final manuscript.

## Supplementary Material

Additional file 1 Bacterial strains and plasmids used in this study. Click here for file

Additional file 2 Primers used in this study. Click here for file

## Figures and Tables

**Table 1:** Regulatory dependence of type III secreted effectors under SPI-1 inducing conditions.

		**A**	**B**	**C**	**D**	**E**	**F**	**G**	**H**	**I**
		wild type	MM+ pWSK29	MM *sprB+*	MM *hilC+*	MM *hilD+*	MM *hilA +*	MM *invF+ sicA +*	MM *ssrB+*	MM *sirA +*

**1**	*sopA-lacZ*	7685 ± 1026	23 ± 1	1 ± 2	0 ± 2	1 ± 2	9 ± 3	**4982 ± 625**	9 ± 3	1 ± 1
**2**	*sopB-lacZ*	10981 ± 922	148 ± 11	85 ± 2	75 ± 8	82 ± 4	199 ± 15	**56160 ± 4842**	**3095 ± 1711**	113 ± 8
**3**	*sopE2-lacZ*	14251 ± 4224	1872 ± 252	2425 ± 748	2477 ± 281	1093 ± 391	3735 ± 65	**31415 ± 1109**	3963 ± 90	2574 ± 69
**4**	*slrP-lacZ*	385 ± 44	305 ± 5	**3511 ± 349**	295 ± 164	448 ± 78	403 ± 87	**1282 ± 138**	308 ± 39	621 ± 23
**5**	*sspHl-lacZ*	796 ± 196	689 ± 26	697 ± 165	668 ± 98	949 ± 103	960 ± 146	**2906 ± 585**	827 ± 165	666 ± 67
**6**	*sspH2-lacZ*	360 ± 34	326 ± 38	412 ± 128	225 ± 16	389 ± 71	453 ± 90	359 ± 24	**15685 ± 1481**	296 ± 11
**7**	*sifA-lacZ*	21664 ± 538	10321 ± 719	9970 ± 528	9232 ± 665	9611 ± 887	10176 ± 817	10329 ± 1153	14085 ± 429	16738 ± 1612
**8**	*sifB-lacZ*	65 ± 2	939 ± 81	1393 ± 849	**19 ± 5**	1537 ± 470	809 ± 150	**30 ± 6**	**14334 ± 2904**	**25 ± 6**
**9**	*sseI-lacZ*	70 ± 4	60 ± 10	69 ± 22	32 ± 2	43 ± 5	61 ± 15	47 ± 10	**5154 ± 1291**	60 ± 15
**10**	*sseG-lacZ*	1931 ± 36	2046 ± 440	1153 ± 257	3001 ± 297	2083 ± 442	2872 ± 314	2444 ± 554	**25454 ± 314**	919 ± 73

The *lac *fusion being measured is listed in the left column. The strain background is listed along the top. Each strain harbors a *lac *fusion to an effector and a plasmid encoding a regulator or the vector control. The values in bold are those showing greater than 3-fold differences compared to the MM + pWSK29 strain that are discussed in the text. Each value represents the mean of at least three experiments ± standard deviation. MM, multi-regulator mutant [Δ(*avrA-invH*)1 *ssrB*::*cat sirA4*::*hyg*].

**Table 2:** Regulatory dependence of type III secreted effectors under SPI-2 inducing conditions.

		**A**	**B**	**C**	**D**	**E**	**F**	**G**	**H**	**I**
		wild type	MM + pWSK29	MM *sprB+*	MM *hilC+*	MM *hilD+*	MM *hilA +*	MM *invF+sicA +*	MM *ssrB+*	MM *sirA +*

**1**	*sopA-lacZ*	1221 ± 35	35 ± 13	24 ± 7	19 ± 7	26 ± 7	48 ± 17	**6129 ± 1152**	75 ± 36	33 ± 6
**2**	*sopB-lacZ*	3573 ± 34	99 ± 5	61 ± 7	61 ± 15	78 ± 10	44 ± 3	**219791 ± 11811**	33 ± 15	46 ± 5
**3**	*sopE2-lacZ*	3530 ± 270	5406 ± 547	4807 ± 538	7285 ± 1182	2347 ± 35	7141 ± 852	**49681 ± 5581**	7141 ± 2059	5222 ± 350
**4**	*slrP-lacZ*	1650 ± 24	1222 ± 92	3091 ± 43	1108 ± 132	1211 ± 163	1688 ± 523	1858 ± 238	1901 ± 311	2146 ± 34
**5**	*sspH1-lacZ*	3744 ± 140	1908 ± 254	1772 ± 471	1686 ± 161	1420 ± 116	1950 ± 128	3558 ± 232	3947 ± 978	1951 ± 154
**6**	*sspH2-lacZ*	6482 ± 1779	764 ± 123	1006 ± 48	655 ± 80	625 ± 84	981 ± 35	850 ± 102	**62305 ± 14432**	890 ± 111
**7**	*sifA-lacZ*	9995 ± 1326	13634 ± 1478	11691 ± 1186	10521 ± 1782	12603 ± 1877	13028 ± 2017	11010 ± 1742	8625 ± 784	21071 ± 1272
**8**	*sifB-lacZ*	2209 ± 503	1085 ± 77	711 ± 89	**49 ± 3**	1264 ± 139	1074 ± 207	**43 ± 6**	**14677 ± 1092**	**43 ± 8**
**9**	*sseI-lacZ*	3543 ± 314	119 ± 23	168 ± 40	130 ± 19	127 ± 32	128 ± 25	108 ± 16	**11049 ± 2830**	165 ± 10
**10**	*sseG-lacZ*	3017 ± 780	1444 ± 93	700 ± 49	1503 ± 109	1146 ± 118	690 ± 121	1083 ± 96	**4229 ± 221**	802 ± 99

The *lac *fusion being measured is listed in the left column. The strain background is listed along the top. Each strain harbors a *lac *fusion to an effector and a plasmid encoding a regulator or the vector control. The values in bold are those showing greater than 3-fold differences compared to the MM + pWSK29 strain that are discussed in the text. Each value represents the mean of at least three experiments ± standard deviation. MM, multi-regulator mutant [Δ (*avrA-invH*)1 *ssrB*::*cat sirA4*::*hyg*].

**Table 3:** Regulation of *sifA *by *ssrAB*.

	LB	MgM
WT *sifA-lac*	5.5 ± 1.6	26.3 ± 4.8
MM *sifA-lac*	3.6 ± 0.5	16.8 ± 2.9
MM *sifA-lac + p-ssrB*	4.8 ± 0.8	29.6 ± 6.9
MM *sifA-lac + *pWSK29	3.8 ± 0.5	15 ± 5.6
Δ ssrA *sifA-lac*	3.8 ± 1.1	14.4 ± 2.7
Δ *ssrB sifA-lac*	3.9 ± 1.4	14.2 ± 2.5
Δ *ssrAB sifA-lac*	2.7 ± 2.4	15.6 ± 2.6

The β-galactosidase activities of strains harbouring a *sifA-lac *fusion were measured using ONPG. Each value represents the mean Miller units of at least three experiments ± standard deviation. WT, wild type 14028; MM, multi-mutant [Δ(*avrA-invH*)1 *ssrB*::*cat sirA4*::*hyg*].
