# Supplementary material for: Systematic analysis of the regulation of type three secreted effectors in Salmonella enterica serovar Typhimurium
Source: BMC Microbiol. 2007 Jan 18;7:3. doi: 10.1186/1471-2180-7-3 (PMC1781944; doi:10.1186/1471-2180-7-3)
Supplement: Additional file 1 — Bacterial strains and plasmids used in this study. [file 1471-2180-7-3-S1.doc]

Additional file 1. Bacterial strains and plasmids used in this study.

| **Strain/Plasmid** | **Relevant characteristics** | **Reference** |
| --- | --- | --- |
| **Strain** |  |  |
| *Escherichia coli* |  |  |
| DH5α | *lacZ*ΔM15 *deoR endA1 gyrA96 hsdR17 recA1 relA1 supE44 thi-1* Δ(*lacZYA-argF*)*U169* | [47] |
| BW20767 | RP4-2-*tet*::Mu-1*kan*::Tn*7* integrant *leu-63*::IS*10 recA1 creC510 hsdR17 endA1 zbf-5 uidA* (Δ*Mlu*I):*pir*^+^*thi* | [58] |
| *S.* Typhimurium |  |  |
| 14028 | Wild type serovar Typhimurium | ATCC^§^ |
| JVR140 | 14028 *sirA4*::*hyg* | [59] |
| MJW129 | 14028 *ssrB*::*cat* | [29] |
| YD038 | 14028 Δ(*avrA-invH*)1 *ssrB*::*cat* *sirA4*::*hyg* | this work |
| YD059 | 14028 Δ(*avrA-invH*)1 *ssrB*::*cat* *sirA4*::*hyg* Φ(*sopA*^+^-*lacZY*^+^) | this work |
| YD060 | 14028 Δ(*avrA-invH*)1 *ssrB*::*cat* *sirA4*::*hyg* Φ(*sopB*^+^-*lacZY*^+^) | this work |
| YD061 | 14028 Δ(*avrA-invH*)1 *ssrB*::*cat sirA4*::*hyg* Φ(*sopD*^+^-*lacZY*^+^) | this work |
| YD062 | 14028 Δ(*avrA-invH*)1 *ssrB*::*cat* *sirA4*::*hyg* Φ(*sopE2*^+^-*lacZY*^+^) | this work |
| YD063 | 14028 Δ(*avrA-invH*)1 *ssrB*::*cat* *sirA4*::*hyg* Φ(*slrP*^+^-*lacZY*^+^) | this work |
| YD064 | 14028 Δ(*avrA-invH*)1 *ssrB*::*cat* *sirA4*::*hyg* Φ(*sspH1*^+^-*lacZY*^+^) | this work |
| YD065 | 14028 Δ(*avrA-invH*)1 *ssrB*::*cat* *sirA4*::*hyg* Φ(*sspH2*^+^-*lacZY*^+^) | this work |
| YD066 | 14028 Δ(*avrA-invH*)1 *ssrB*::*cat* *sirA4*::*hyg* Φ(*sifA*^+^-*lacZY*^+^) | this work |
| YD067 | 14028 Δ(*avrA-invH*)1 *ssrB*::*cat* *sirA4*::*hyg* Φ(*sifB*^+^-*lacZY*^+^) | this work |
| YD068 | 14028 Δ(*avrA-invH*)1 *ssrB*::*cat* *sirA4*::*hyg* Φ(*sseI*^+^-*lacZY*^+^) | this work |
| YD069 | 14028 Δ(*avrA-invH*)1 *ssrB*::*cat* *sirA4*::*hyg* Φ(*sseG*^+^-*lacZY*^+^) | this work |
| YD435 | 14028 Δ*invF*::*cat* Φ(*slrP*^+^-*lacZY*^+^) | this work |
| YD436 | 14028 Δ*sicA*::*cat* Φ(*slrP*^+^-*lacZY*^+^) | this work |
| YD520 | 14028 Δ*sprB*::*cat* Φ(*slrP*^+^-*lacZY*^+^) | this work |
| YD493 | 14028 Φ(*sifA*^+^-*lacZY*^+^) | this work |
| YD522 | 14028 Δ*ssrA*::*cat* Φ(*sifA*^+^-*lacZY*^+^) | this work |
| YD523 | 14028 Δ*ssrB*::*cat* Φ(*sifA*^+^-*lacZY*^+^) | this work |
| YD524 | 14028 Δ*ssrAB*::*cat* Φ(*sifA*^+^-*lacZY*^+^) | this work |
| **Plasmids** |  |  |
| pCR2.1 TOPO | Ap^r^ Km^r^, ColE1 | Invitrogen |
| pVIK112 | Km^r^, ori R6K, *lacZY* for transcriptional fusion | [54] |
| pRE112 | Cm^r^, ori R6K, *sacB* | [52] |
| pWSK29 | Ap^r^, pSC101 ori | [60] |
| pMJW120 | Ap^r^, pACYC ori, *ssrB* | [29] |
| pTopoT-YD19 | Ap^r^ Km^r^, ColE1, up*-*ttss1* | this work |
| pTopoT-YD20 | Ap^r^ Km^r^, ColE1, *ttss1*-dw^**^ | this work |
| pKD3 | FRT*-cat-*FRT, oriR6K | [50] |
| pKD4 | FRT*-kan-*FRT, oriR6K | [50] |
| pKD46 | Ap^r^ P_BAD_ *gam bet exo* pSC101 oriTS | [50] |
| pCP20 | Ap^r^ Cm^r^ *cI857* λP_R_ *flp* pSC101 oriTS | [56] |
| pCE36 | Km^r^, ori R6K, FRT-*lacZY* | [55] |
| pYD15 | Ap^r^, pSC101 ori, *sprB* | this work |
| pYD16 | Ap^r^, pSC101 ori, *hilC* | this work |
| pYD17 | Ap^r^, pSC101 ori, *hilD* | this work |
| pYD23 | Ap^r^, pSC101 ori, *invF* | this work |
| pYD24 | Ap^r^ Km^r^, ColE1, up*- *ttss1*-dw** | this work |
| pYD25 | Cm^r^, ori R6K, up*- *ttss1*-dw** | this work |
| pYD27 | Ap^r^, pSC101 ori, *ssrB* | this work |
| pYD28 | Ap^r^, pSC101 ori, *sirA* | this work |
| pYD29 | Ap^r^, pSC101 ori, *hilA* | this work |
| pYD38 | Ap^r^, pSC101 ori, *sicA* | this work |
| pYD40 | Ap^r^, pSC101 ori, *invF*-*sicA* | this work |
| pYD56 | Tet^r^, pACYC ori, P*sifA*-*luxCDABE* | this work |
| pSB384 | Tet^r^, pACYC ori, promoterless *luxCDABE* | [57] |

§, American Type Culture Collection; ttss1, SPI1 region encoding a TTSS; *, sequence upstream of the TTSS1 encoding region of SPI1; **, sequence downstream of the TTSS1 encoding region of SPI1.
