# Supplementary material for: Systematic analysis of the regulation of type three secreted effectors in Salmonella enterica serovar Typhimurium
Source: BMC Microbiol. 2007 Jan 18;7:3. doi: 10.1186/1471-2180-7-3 (PMC1781944; doi:10.1186/1471-2180-7-3)
Supplement: Additional file 2 — Primers used in this study. [file 1471-2180-7-3-S2.doc]

**Additional file 2. Primers used in this study.**

| **Name** | **Usage/Sequence** | **Binding site** |
| --- | --- | --- |
|  | **Deletion of SPI1** |  |
| BA1093 | 5’caggcgctcaccttcttccg | *invH* |
| BA1094 | 5’gccatttcccagctttcgctgattca | *stm2902* |
| BA1095 | 5’*gagctc*gtcgtcatgcaaaccttactgttca, *Sac*I site in italic | *sitC* |
| BA1096 | 5’*actagt*catactcaaggcgcacaacgatt, *Spe*I site in italic | *avrA* |
|  | **Cloning of regulator genes** |  |
| BA1097 | 5’ccaaccacctccggatgatta | 3’ of *sprB* |
| BA1098 | 5’cattaccgaagctgtggtcagtt | 5’ of *sprB* |
| BA955 | 5’cctgggcgactactgcgcaa | 3’ of *hilC* |
| BA1099 | 5’tgagttccttatagcacacaggataa | 5’ of *hilC* |
| BA947 | 5’gccggaaggagatagtgtca | 3’ of *hilD* |
| BA1100 | 5’cagtaggataccagtaaggaacat | 5’ of *hilD* |
| BA1008 | 5’tgcatcaggccaagatcggttgaac | 3’ of *hilA* |
| BA1101 | 5’ttcaccctgtaagagaatacactatt | 5’ of *hilA* |
| BA1102 | 5’cagcactctggccaaaagaat | 3’ of *invF* |
| BA1103 | 5’cccgctcccggtattgttta | 5’ of *invF* |
| BA1260 | 5’caggagtaagtaatggattatca | 5’ of *sicA* |
| BA1261 | 5’ctacttgcgtcatttaccataat | 3’ of *sicA* |
| BA179 | 5’actatcagtagcgttatccctattc | 5’ of *sirA* |
| BA180 | 5’gccaggttgctgcgaaagtagctgg | 3’ of *sirA* |
|  | ***lacZ* fusion to effector genes** |  |
| BA1049 | gctgctttcctcccaagattca | *sopA* |
| BA1050 | 5’atgcgggttgaggctggacta | 3’ of *sopA* |
| BA257 | 5’*gaattc*ctgttcaagcatggaataggaaaaacg, *Eco*RI site in italic | *sopB* |
| BA258 | 5’*gaattc*gaagatcgcgctgtaagttatagagg, *Eco*RI site in italic | 3’ of *sopB* |
| BA1051 | 5’ggaccatgcgctggaagtgtta | *sopD* |
| BA1052 | 5’gcacccatctttaccaatgtgcaaa | 3’ of *sopD* |
| BA1055 | 5’cataacactatccacccagcacta | *sopE2* |
| BA1056 | 5’tcaggaggcattctgaagatactt | 3’ of *sopE2* |
| BA1057 | 5’ggcgttcggactggaagataa | 5’ of *sspH1* |
| BA1058 | 5’gcggtgaatatcgtgctcagtt | 3’ of *sspH1* |
| BA1059 | 5’cacctggtgcatcagttacga | *sspH2* |
| BA1060 | 5’gcgaggaccgggtcacattt | 3’ of *sspH2* |
| BA1061 | 5’agaatacccgacacccggattt | 5’ of *slrP* |
| BA1062 | 5’gcgttcagctatcgccagta | 3’ of *slrP* |
| BA1063 | 5’gtctgagaaagcgtcgtctgat | 3’ of *sifA* |
| BA1064 | 5’ccgatacctttagctgtgaagt | *sifA* |
| BA1065 | 5’gcaaagatccactgccgatcaa | *sifB* |
| BA1066 | 5’tcaactctggtgatgagcctca | 3’ of *sifB* |
| BA1067 | 5’ggaggatgtggtcagcagattt | *sseI* |
| BA1068 | 5’cctccacggtgcgcttacatt | 3’ of *sseI* |
| BA1088 | 5’catctcggggagaaccatgaaa | *sseG* |
| BA1089 | 5’attactccggcgcacgttgtt | 3’ of *sseG* |
| BA1388 | *aacaacaaagcggctgtttatgttgttttttataaaatca*gtgtaggctggagctgcttc | 3’ of *sifA* (italic), pKD4 |
| BA1389 | *gtacgtgagtaaaccctgaacgtgacgtctgagaaagcgt*catatgaatatcctccttag | 3’ of *sifA* (italic), pKD4 |
|  | **Deletion of regulator genes** |  |
| BA1321 | 5’*agcatggtttatacagacgtgttccgcgcaaaagctgcat*gtgtaggctggagctgcttc | *invF* (italic), pKD3 |
| BA1322 | 5’*gcacatgccagcactctggccaaaagaatatgtgtcttca*catatgaatatcctccttag | *invF* (italic), pKD3 |
| BA1323 | 5’*taagttctacaaagtcggtgacagataacaggagtaagta*gtgtaggctggagctgcttc | *sicA* (italic), pKD3 |
| BA1324 | 5’*ccgctacggctaatgctacttgcgtcatttaccataatta*catatgaatatcctccttag | *sicA* (italic), pKD3 |
| BA1290 | *cgttaaaacgtgtaactcattaactgcacttttgcattcc*gtgtaggctggagctgcttc | *sprB* (italic), pKD3 |
| BA1291 | *gtgcgctgtaaatgcgcgtctagtttcagtccccggaaca*catatgaatatcctccttag | *sprB* (italic), pKD3 |
| YD68 | *tgtcacaggcgattctatcattcggattttccgataaatt*gtgtaggctggagctgcttc | *ssrA* (italic), pKD3 |
| YD69 | *ggaaaatcttgctattaatgaccatatcatctgtcgccag*catatgaatatcctccttag | *ssrA* (italic), pKD3 |
| YD70 | *atcttaattttcgcgagggcagcaaaatgaaagaatataa*gtgtaggctggagctgcttc | *ssrB* (italic), pKD3 |
| YD71 | *aatatgaccaatgcttaataccatcggacgcccctggtta*catatgaatatcctccttag | *ssrB* (italic), pKD3 |
|  | **Construction of the P*sifA*-*lux* reporter plasmid** |  |
| YD1 | 5’ atcgatgcgcaacgctaacaaatccaca | Upstream of PsifA promoter |
| YD2 | 5’ gaattcgcgaagaataatggtggcatcat | *sifA* |
